# Assessment of Knowledge and Attitudes Toward Organ Donation in the Qassim Region, Saudi Arabia

**DOI:** 10.7759/cureus.77768

**Published:** 2025-01-21

**Authors:** Abdullah M Althunayyan, Abdulaziz H Alsadun, Fahad Alrumayh, Lamees Alsulaim

**Affiliations:** 1 Department of Surgery, College of Medicine, Qassim University, Buraydah, SAU

**Keywords:** attitudes, knowledge, organ donation, qassim region, transplant

## Abstract

Introduction: Organ donation is the transplantation of tissues or organs from one person to another, and it is considered a method to save lives when a patient has end-stage organ failure. Community willingness to engage in organ donation programs is imperative in promoting successful organ transplantation.

Methods: This research employed a cross-sectional study design involving 420 adult participants from the Qassim Region, Saudi Arabia. An online questionnaire was used to collect data and later analyzed using IBM SPSS Statistics for Windows, Version 27 (Released 2020; IBM Corp., Armonk, New York, United States).

Results: The results reveal that only six (1.4%) participants had donated an organ before. One hundred sixty-eight (40.0%) participants believed that a person could donate their organ during their lifetime, with 225 (53.6%) aware that kidneys could be donated. A total of 198 (47.1%) participants supported the practice of organ donation. Religious motivation was the primary reason for the majority of participants willing to donate their organs (128, 64.7%). Conversely, among those unwilling to donate, 86 individuals (38.7%) cited a lack of knowledge about the practice as their reason. Two hundred sixty-six (63.3%) respondents expressed willingness to donate organs if laws and religion encouraged the practice. One hundred fifteen (27.4%) had a low knowledge level, 203 (48.3%) had a moderate knowledge level, and 102 (24.3%) had a high knowledge level. Age, education level, and occupation showed a statistically significant relationship with the knowledge score on organ donation (p < 0.05).

Conclusion: The study revealed that the general population exhibited a moderate understanding of and favorable attitude toward organ donation. Age, education level, and occupation significantly influenced knowledge scores related to organ donation. Additionally, religious beliefs emerged as the primary driver for organ donation. There is a need to raise public knowledge about the importance of organ donation in saving lives, improving quality of life, and establishing sustainable and ethical organ transplantation systems in Saudi Arabia.

## Introduction

Organ donation refers to the grafting of an organ from a living or brain-dead human being to a living patient with end-stage organ failure [1]. As the prevalence of chronic diseases increases and more advancements in medicine are realized, organ transplantation has become increasingly common as a life-saving intervention. In 2022, more than 157,000 organ transplants were performed worldwide, with the kidney being the most common, followed by the liver [2]. In Saudi Arabia, 2,091 organ transplants were conducted in 2023 from both living and deceased donors [3]. Despite the critical role of organ donation, there remains a significant gap between the number of available organs and the required organs. While the demand for organ transplants is increasing, the supply of organ donors is not keeping pace with this growing need [4]. A lack of adequate organs for transplantation leads to preventable deaths, which highlights the need for organ donation.

Attitudes toward organ donation are influenced by a combination of religious beliefs, cultural values, and awareness about the benefits and ethical considerations associated with organ donation [5]. Soqia et al. determined that religious beliefs have a particularly negative effect on opinions and attitudes toward organ donation, with many spiritual people viewing it as unacceptable against the will of God [6]. However, as Tanna et al. note, organ donation will only become increasingly necessary, and there is a need for more people to be adequately informed and appreciate it better [7]. Exceptionally, donation after brainstem death (DBD) can be significant in closing the existing deficit. In Saudi Arabia, 14,139 possible DBD donors were reported to the Saudi Center for Organ Transplantation (SCOT) between 1986 and 2020; 11,099 were male, while 3,040 were female [8].

To boost the number of organ donors, citizens need a thorough understanding of and a favorable outlook on organ donation and transplantation. To boost the number of organ donors, citizens must possess a detailed knowledge of and a favorable outlook on organ donation and transplantation. Presently, there needs to be more comprehensive research on the Saudi Arabian public's understanding and attitudes regarding organ donation. So the objective of this study is to evaluate the knowledge levels, attitudes, and motivations related to organ donation among adults in the Qassim Region, with a focus on identifying demographic and cultural factors that influence these perceptions.

## Materials and methods

Study design and duration

This study adopted a cross-sectional design. It was conducted from April 5, 2024 to August 7, 2024 among adults in the Al-Qassim region of the Kingdom of Saudi Arabia (KSA). It aimed to evaluate the knowledge and attitudes regarding organ donation.

Inclusion and exclusion criteria

The inclusion criteria involved all male and female individuals aged 18 and above who were residents of the Al-Qassim region, regardless of their nationalities. Informed consent was required from participants to participate in the study.

The exclusion criteria included individuals who had previously donated an organ since their history could affect the study's variables. Additionally, individuals working with an organ donation center were excluded to prevent potential bias due to their professional backgrounds. Further exclusions included those with severe psychiatric disorders, terminal illnesses, or specific chronic diseases that could interfere with their ability to participate fully.

Sampling technique

This study used convenience sampling, selecting participants based on their availability and willingness to participate during the research period by sending the questionnaires online through platforms like WhatsApp and healthcare institutions to ensure most of the Qassim region cities were involved.

Sample size

The Cochran sample size formula was applied to determine the sample size. The formula follows:

$n = \frac{Z^2 p (1 - p)}{d^2}$ where n is the sample size, Z denotes the critical value for 95% CI, 50% is the predetermined proportion, and d is the margin of error (5%) [9]. Based on these parameters, the initial minimum sample size calculated was 384. However, a slightly larger sample size of 420 respondents was selected to enhance the study's reliability.

Data collection tools and procedure

Data was gathered using a prior study's structured and validated electronic questionnaire [10], which was used to assess the knowledge about organ donation in the Jazan region. The questionnaire comprised three main sections: the first gathered sociodemographic data, the second assessed participants' knowledge about organ donation, and the third evaluated participants' attitudes. A pilot study that involved nearly 10% of the participants was conducted to assess the questionnaire's readability and understandability. Individuals from the pilot study were excluded from the main study. The internal consistency was evaluated using Cronbach's alpha, which resulted in a value of 0.845, which indicated a good level of internal consistency. The final questionnaire, incorporating feedback from the pilot study, was then disseminated through a structured, validated electronic questionnaire via a link through social platforms and through health institutions. Then the result was classified as low knowledge with 6 points and less, moderate 7-9 points, and excellent knowledge above 10 and above.

Missing data were excluded from the analysis, with no imputation performed for incomplete responses.

Statistical analysis

Following data collection, the responses were manually reviewed and coded into a Microsoft Excel sheet before being entered into and analyzed with IBM SPSS Statistics for Windows, Version 27 (Released 2020; IBM Corp., Armonk, New York, United States). The analysis involved both descriptive and comparative statistics. Descriptive statistics included frequencies and percentages for categorical variables. The knowledge levels toward organ donation were assessed using a scoring system based on participants' total scores and classified participants into low, moderate, and high knowledge categories based on their total scores. The Chi-square test was employed to examine associations between sociodemographic factors and the knowledge score toward organ transplantation, with a p-value less than 0.05 considered statistically significant.

Ethical considerations

The Committee of Research Ethics of Qassim University approved the study before commencing data collection (24-84-19) on April 5, 2024. All data gathered were anonymized by omitting personal identifiers, ensuring that individual identities could not be connected to the results. Participants were thoroughly briefed on the study's objectives, methodology, possible risks, and expected benefits before providing voluntary and uninfluenced consent.

## Results

A total of 420 participants were involved in the study. Table 1 reveals that the majority (218, 51.9%) of the participants were aged 18-30 years, with more than half (215, 51.2%) of them being males. The vast majority (402, 95.7%) of the respondents were Saudi nationals, with the majority (348, 82.9%) of them with a university education. More than half (235, 56.0%) of them were employees, with a notable proportion of 159 (37.9%) of them being Buraydah residents and a substantial proportion of 172 (41.0%) of them with a monthly income of less than 5000 SAR.

**Table 1:** Socio-demographic information of the participants (n = 420) Frequencies (n) and proportion (%) used to present data

Socio-demographic information	Category	Frequency and proportion n (%)
Age	18-30 years	218 (51.9%)
	31-40	86 (20.5%)
	41-50	68 (16.2%)
	Older than 50 years	48 (11.4%)
Gender	Male	215 (51.2%)
	Female	205 (48.8%)
Nationality	Saudi	402 (95.7%)
	Non-Saudi	18 (4.3%)
Education level	Primary school	6 (1.4%)
	Intermediate school	3 (0.7%)
	High school	63 (15.0%)
	University education	348 (82.9%)
Job	Student	124 (29.5%)
	Unemployed	49 (11.7%)
	Employee	235 (56.0%)
	Retiree	12 (2.9%)
Residency	Al-Badyea	57 (13.6%)
	Al-Rass	35 (8.3%)
	Authal	39 (9.3%)
	Bukayriyah	16 (3.8%)
	Buraydah	159 (37.9%)
	Riyadh al Khabra	23 (5.5%)
	Unaizah	45 (10.7%)
	Uyun Al-Jawa	46 (11.0%)
Monthly income	Less than 5000 SAR	172 (41.0%)
	5000-10000 SAR	66 (15.7%)
	10000-15000 SAR	96 (22.9%)
	More than 15000 SAR	86 (20.5%)

Table 2 revealed that only six (1.4%) participants had donated an organ before, and nine (2.1%) had worked in an organ donation center. A notable proportion of 168 (40.0%) of the participants believed that an individual can donate their organs during their lifetime. The majority (225, 53.6%) were informed that the kidney can be donated, followed by the liver, which was reported by 84 (20.0%), while the heart was reported by 47 (11.2%) participants. Only 24 (5.7%) participants reported that lungs can be donated. Interestingly, nine (2.1%) participants did not know that organs could be donated. The majority (327, 77.9%) omit knowing about the existence of official agencies and centers for organ donation in KSA. Among them, nearly one-third (95, 29.1%) were familiar with the legal framework involved.

**Table 2:** Assessment of participants’ knowledge regarding organ donation Data presented as frequencies (n) and proportion (%) KSA: Kingdom of Saudi Arabia

Questions	Categories	Frequency and proportion n (%)
Have you donated organ before?	Yes	6 (1.4%)
	No	414 (98.6%)
Do you work in organ donation center?	Yes	9 (2.1%)
	No	411 (97.9%)
If you are open to donating your organs, when would you prefer to make this donation?	When alive	168 (40.0%)
	Only after my brain death	144 (34.3%)
	After death	51 (12.2%)
	Uncertain	57 (13.5%)
Which organs do you believe are eligible for donation?	Kidneys	225 (53.6%)
	Liver	84 (20.0%)
	Heart	47 (11.2%)
	Eyes	31 (7.4%)
	Lungs	24 (5.7%)
	I don’t know	9 (2.1%)
Do you know about the existence of official centers and agencies in KSA for organ donation?	Yes	327 (77.9%)
	No	93 (22.1%)
Do you know about the regulations and legal aspects related to brain death and organ donation or transplantation in KSA?	Yes	95 (29.1%)
	No	232 (70.9%)

Table 3 depicts the participants’ attitudes toward organ donation. A substantial proportion (198, 47.1%) of the participants were in agreement with the practice of organ donation. When considering the timing of organ donation, the majority (232, 55.2%) preferred to donate after their death. Furthermore, the majority (306, 72.9%) of them would like to donate their organ to both their relatives and non-relatives. The main motivation behind the willingness to donate was religious reasons, as depicted by 128 (64.7%) participants. Conversely, of those not willing to donate their organs, a notable proportion (86, 38.7%) cited a lack of adequate knowledge. Moreover, the majority (266, 63.3%) of the respondents were willing to give organs if laws and religion encouraged it, with more than half (226, 53.8%) of them expressing their desire and need to promote organ donation. However, only 40 (9.5%) respondents expressed reservations and hesitation about the need to promote organ donation, with the majority (23, 57.5%) of them citing the possibility of encouraging organ trafficking.

**Table 3:** Participants' attitude assessment toward organ donation Participants’ attitude assessment toward organ donation presented in frequencies (n) and proportion (%)

Questions	Categories	Frequency and proportion n (%)
What is your stance on organ donation?	I support it	198 (47.1%)
	I oppose it	30 (7.1%)
	I am unsure	70 (16.8%)
	I am neutral	122 (29.0%)
When would you prefer to donate your organs if you choose to do so?	Only while I am alive	51 (12.1%)
	After I have passed away	232 (55.2%)
	I do not have a preference	137 (32.7%)
If you are open to donating your organs, who would you choose to donate them to?	My relatives only	108 (25.7%)
	Non-relatives only	6 (1.4%)
	Both relatives and non-relatives	306 (72.9%)
If you are willing to donate your organs, what motivates you?	Religious reasons	128 (64.7%)
	Social reasons	7 (3.5%)
	A need to help people	44 (22.2%)
	The recipient's age	3 (1.5%)
	The recipient's health condition	13 (6.6%)
	Financial reasons	3 (1.5%)
If you choose not to donate your organs, what are your reasons?	No financial incentives	10 (4.5%)
	Family and social obstacles	19 (8.6%)
	Fear of not getting proper medical care	9 (4.1%)
	Discomfort discussing death	14 (6.3%)
	Lack of knowledge	86 (38.7%)
	Religious beliefs	69 (31.1%)
	The organ receiver choice is unfair	15 (6.7%)
If religious and legal frameworks supported organ donation, would you consider donating?	Yes	266 (63.3%)
	No	29 (6.9%)
	I’m not sure	125 (29.8%)
What are your thoughts on advocating for organ donation?	I believe it is necessary	226 (53.8%)
	I believe it is not necessary	40 (9.5%)
	Not sure	154 (36.7%)
If you believe promoting organ donation is unnecessary, what are your reasons?	Concern about organ trafficking	23 (57.5%)
	Fear that donated organs may be mishandled or wasted	4 (10.0%)
	Religious beliefs	8 (20.0%)
	Other reasons (such as post-operative pain, scarring, body disfigurement, or fear of family rejection)	2 (5.0%)
	No reasons were specified	3 (7.5%)

Table 4 below shows that among the participants, 115 (27.4%) were classified with low knowledge (total score below 50%, scoring 6 or fewer points), 203 (48.3%) had moderate knowledge, and 102 (24.3%) demonstrated high knowledge (total score above 75%, scoring 10 or more points).

**Table 4:** Participants’ knowledge level scores of organ donation Participants’ knowledge level scores of organ donation are presented in frequencies (n) and proportion (%)

Knowledge level	Total scores (%)	Total scores (n)	Percentage score
Low knowledge level	<50%	6 or fewer points	27.4%
Moderate knowledge level	50%-75%	Between 7 and 9 points	48.3%
High knowledge level	>75%	10 or more points	24.3%

Table 5 provides the relationship between socio-demographic information and the knowledge score toward organ donation among the participants. A statistically significant association between age, education level, a job with p-values (<0.001, 0.003, <0.001), and the knowledge score toward organ donation was observed. However, there was no statistically significant association between gender, nationality, residency, monthly income, and the knowledge score toward organ donation with p-values greater than 0.05. The study noted that younger participants, those aged 18-30 years, had notably higher knowledge scores than those in other age categories (p < 0.001). Similarly, individuals with a university education and those who were employed achieved higher scores compared to their peers.

**Table 5:** The association between socio-demographic information and the knowledge score toward organ transplantation Association between socio-demographic variables and knowledge score toward organ donation *ANOVA test used to determine significance. Significance at p < 0.05 level

Variables	Category	Mean ± STD	F-value	p-value
Age	18-30	8.13 ± 2.16	14.1	<0.001*
	31-40	7.25 ± 2.41		
	41-50	7.61 ± 2.78		
	>50	7.37 ± 2.29		
Gender	Male	7.76 ± 2.39	0.52	0.472
	Female	7.52 ± 2.42		
Nationality	Saudi	7.16 ± 2.52	0.20	0.653
	Non-Saudi	7.03 ± 2.29		
Education level	Primary school	6.03 ± 2.95	4.75	0.003*
	Intermediate school	6.17 ± 2.45		
	High school	7.53 ± 2.35		
	University education	8.64 ± 2.04		
Job	Student	7.41 ± 2.31	11.4	<0.001*
	Unemployed	7.39 ± 2.69		
	Employee	8.12 ± 2.13		
	Retiree	7.74 ± 2.29		
Residency	Al-Badyea	7.36 ± 2.53	0.45	0.832
	Al-Rass	7.64 ± 2.46		
	Authal	7.55 ± 2.31		
	Bukayriyah	7.41 ± 2.70		
	Buraydah	7.89 ± 2.17		
	Riyadh al Khabra	7.53 ± 2.19		
	Unaizah	7.61 ± 2.42		
	Uyun Al-Jawa	7.60 ± 2.37		
Monthly income	Less than 5000 SAR	8.16 ± 2.34	0.62	0.534
	5000-10000 SAR	7.35 ± 2.51		
	10000-15000 SAR	7.79 ± 2.48		
	More than 15000 SAR	7.81 ± 2.32		

## Discussion

This study focuses on a region-specific population, providing valuable localized insights into organ donation knowledge and attitudes in Qassim. Most respondents were young adults aged 18-30 years, with a predominance of males. The study revealed that only six (1.4%) participants had donated an organ before, and nine (2.1%) had worked in an organ donation center. The results revealed a moderate level of knowledge about organ donation, with 48.3% of participants scoring between 7 and 9 out of a possible 14 points, yielding a mean score of 7.52 and a standard deviation of 2.56. This finding can be attributed to a higher proportion of young adult participants; most had a university education level and were likely to access information readily [11]. A substantial proportion of 168 (40.0%) participants believed that a person could donate their organ during their lifetime. Most participants expressed knowledge about the organs donated, with the remaining 225 (53.6%) reporting kidney. Similar to our findings, Alnasyan et al. found that the kidney is the most widely recognized organ for donation [12]. Among the participants, 327 (77.9%) were aware of the organ donation official agencies and centers in Saudi Arabia, whereas only 95 (29.1%) were familiar with the legal framework concerning organ donation. This finding is in line with the study by AlHarthi and Alzahrany, which reported that while most Saudi adults were aware of the donation centers, they lacked knowledge about the legal aspects related to organ donation [13]. The findings underscore the need for a coordinated effort to enlighten the public on the policies and processes of organ donation [14].

A substantial proportion of 198 (47.1%) participants agreed with organ donation, with more than half of the participants (232, 55.2%) expressing readiness to donate their organs after death. A comparable study conducted by Ibrahim and Randhawa in Nigeria reported that most of the participants expressed a desire for organ donation upon their death [15]. A significant majority (306, 72.9%) of the respondents would be willing to donate their organs to relatives and non-relatives, with the majority (128, 64.7%) being motivated by religious reasons. This is in line with the findings of Islam, which observed that the Islamic faith urges individuals to save lives and help one another, as stated in the Sunna and the Holy Quran [16]. Of those not willing to donate their organs, a notable proportion (86, 38.7%) lacked knowledge about the practice. The study noted that participants aged 18-30 showed significantly higher knowledge scores than other age groups. Participants with a university education had better knowledge scores compared to those with less educational attainment. This aligns with a study by Khushaim et al. in Jeddah, Saudi Arabia, which found that university-educated young adults had better knowledge [17]. Furthermore, employed participants showed significantly higher knowledge scores than those in other occupational categories.

Regarding attitude, the majority (266, 63.3%) of the respondents expressed willingness to donate organs if laws and religion encouraged it, while more than half (226, 53.8%) expressed their desire and need to promote organ donation. Our findings align with the study by Alharbi and Alenzi, which reported a positive attitude toward organ donation among most adults in Hafar Al-Batin City, Saudi Arabia [18]. Only 40 (9.5%) respondents expressed reservations and hesitation about the need to promote organ donation; most (23, 57.5%) of them cited the possibility of encouraging organ trafficking.

This study's drawback was using a cross-sectional study methodology, which can only identify relationships between attributes but not causalities. Given that the study used online questionnaires, it relied on respondents accurately documenting their responses with no method to verify this, which could have resulted in bias. Furthermore, because the study was conducted in only one region, Qassim, the results cannot be generalized to the entire Saudi Arabian population by using validated questionnaires and/or interviewing the participants as well as the importance of community activities about organ donation and the importance of saving lives.

## Conclusions

The study revealed a moderate level of knowledge and a favorable attitude toward organ donation among the general population of the Qassim region in Saudi Arabia. Age, education level, and job were significantly associated with the knowledge score toward organ donation. The Islamic religion was found to be the primary motivation for organ donation. The study observed willingness and desire for organ donation among the general population of the Qassim region in Saudi Arabia. There is a need to raise public knowledge about the value of organ donation in saving lives, improving quality of life, and establishing sustainable and ethical organ transplantation systems in Saudi Arabia.
